# Transgene Flow: Challenges to the On-Farm Conservation of Maize Landraces in the Brazilian Semi-Arid Region

**DOI:** 10.3390/plants11050603

**Published:** 2022-02-23

**Authors:** Gabriel Bianconi Fernandes, Ana Cláudia de Lima Silva, Maitê Edite Sousa Maronhas, Amaury da Silva dos Santos, Paola Hernandez Cortez Lima

**Affiliations:** 1 National Agroecology Coalition Biodiversity Working Group, Viçosa 36570-972, Brazil; 2 Goiás Federal Institute, 76600-000 Goiânia, Brazil; ana.claudia@ifg.edu.br; 3 Brazilian Semiarid Articulation, Recife 52050-355, Brazil; maronhas@gmail.com; 4 Embrapa Tabuleiros Costeiros, Aracaju 49025-040, Brazil; amaury.santos@embrapa.br; 5 Embrapa Alimentos e Territórios, Maceió 57036-000, Brazil; paola.cortez@embrapa.br

**Keywords:** coexistence, GMO monitoring, *Zea mays* L., community seed banks, family farming

## Abstract

Brazil is one of the largest global producers of genetically modified crops and a center of origin and diversification of relevant species for agriculture and food. Transgenic monocultures occupy around 50 million hectares, whereas smallholder farmers, indigenous people, and traditional communities are responsible for in-situ/on-Farm conservation of local genetic resources. Based on 15 years of expertise in regulating GMOs and in cross-institutional agrobiodiversity conservation projects, this article discusses the challenges regarding the coexistence of these two agricultural models based on transgene flow detection in maize landraces. As part of a broad and unique participatory transgene-flow-monitoring process, 1098 samples of maize landraces were collected in the Brazilian Semi-arid Region between 2018 and 2021 and analyzed using immunochromatographic strips. The tests revealed 34% of samples with presence of GM proteins. It is concluded that the biosafety standards in force in Brazil do not allow the assurance of on-Farm conservation of maize. The sectors that contribute to agrobiodiversity conservation and do not benefit from using GM seeds are taking on the burden of this process. Transgene flow can be reduced by approving and enforcing more effective coexistence rules that consider maize landraces crop areas also as seed-producing areas added to full disclosure of commercial seeds origin.

## 1. Introduction

The risks of transgene flow into landraces and their wild relatives have been discussed since the mid-1990s with the first commercial releases of GM maize varieties [1,2,3]. This unintended presence of transgenes in landraces has had effects on the economy [4,5,6], the conservation of the species’ diversity [7,8], farmers’ rights [9,10], and their sociocultural dynamics [5,11]. Over the last 20 years, studies carried out in different countries have identified the unintentional presence of modified genes in landraces conserved in the field by farmers [12,13,14,15,16,17,18]. The presence of GM maize grains and/or pollen outside the areas for which these plants were designed becomes a matter of special concern in regions considered to be centers of origin and diversity of the species [2,8].

The vast Brazilian territory is home to thousands of landraces of maize grouped into at least 23 breeds and two racial complexes [19]. Recent evidence obtained from cross-referencing linguistic, migration, and archaeological data suggests that maize migrated from the Mexico region, not yet fully domesticated, and was selected and adapted to other territories over millennia through intentional human action [20]. One of the territories of this co-evolution is the Brazilian Amazon. The breed locally known as “entrelaçado” is endemic to the region and is home to a great diversity of landrace varieties [21].

Studies in other regions of the country reveal the presence of a great diversity of landraces [22,23], wild relatives, and endemic varieties [21,24], suggesting the recognition of microcenters of species diversity [25,26,27]. These areas correspond to territories with a strong presence of family farmers, indigenous peoples, and traditional communities [28], a fact that reinforces the central role of local communities in on-Farm dynamic conservation and their importance for the evolution and adaptation of the species, for instance, facing climate change [29,30]. Available studies on transgene flow into landraces reinforce the relevance of the topic for the future of on-Farm conservation. However, in general, these are carried out in a few locations in order to measure the conditions and distances in which the gene flow occurs between GM and non-GM maize in addition to confirming the unintentional presence of transgenes in non-GM plants. An assessment of the phenomenon on a larger scale, embedded in the reality of family farmers, has not yet been carried out.

The Brazilian Semi-arid Region (SAB) is located in the northeast of the country and in the north of the state of Minas Gerais. It covers an area equivalent to 12% of the national territory, housing 28 million inhabitants. Its predominant climate is of the BSh (Köppen) type, considered tropical hot and humid with autumn-winter rains, with average annual precipitation lower than 800 mm, marked by irregular rainfall and high rates of evapotranspiration. The predominant biome is the Caatinga, made up especially of legumes, grasses, *Euphorbiaceae*, bromeliads, and cacti.

In this region, given its history, the civil society made an organized effort to include in the national agenda issues that are specific to the region, thus creating the Brazilian Semi-arid Articulation (Articulação do Semiárido Brasileiro—ASA) in 1999, initially comprising 61 civil society organizations and union federations that denounced the intentional impoverishment of the local population associated with droughts, hunger, land, and water concentration in private lands as well as public policies that have historically strengthened the aforementioned elements. ASA then began to demand a policy suited to the reality of the region based on the concept of “Coexistence with the Semi-arid” as a way to overcome the politically dominant logic of combating drought [31].

The Semi-arid Agrobiodiversity Management Program (2015–2018) is the result of previous actions to reclaim and conserve landraces, supported by the Catholic Church’s Base Ecclesiastical Communities. During the execution of this Program, approximately 1000 houses and community seed banks were supported, where family farmers collectively store their seeds for the purpose of use, conservation, exchange, and commercialization, directly mobilizing 20,000 families. More than 700 landraces of *Phaseolus vulgaris*, *P. lunatus,* and *Vigna* spp. and more than 400 varieties of maize (*Zea mays* L.), among others, were identified.

Based on the information and demands generated by the Program, another project was created as a partnership between the Brazilian Agricultural Research Corporation (Embrapa) and the ASA, called Agrobiodiversity of the Semi-arid. The project was launched in 2019 with the purpose of strengthening and enhancing the farming families’ strategies of coexistence with the semi-arid region through the use, conservation, and valuation of socioagrobiodiversity based on coordinated actions with local socio-technical networks, supporting public policies and promoting knowledge sharing and creation in innovative agroecological processes. It covers more than 2600 farming families and 147 community seed banks.

During these actions, the imminent transgene flow into maize landraces, the risk of genetic erosion, and the reduction of agrobiodiversity were identified. Such facts enabled the implementation of large-scale monitoring of transgene flow into maize landraces in the communities of family farmers who benefit from the two aforementioned projects in order to verify its occurrence and frequency to provide farmers with guidance and to subsidize public policies and further studies. This emergent issue also called into question current maize coexistence rules, limited to establish a 100-m isolation distance between GM and non-GM fields or 20 m when 10 rows of conventional maize are added (see Section 3.3).

Monitoring was carried out through rapid immunochromatographic tests, popularly known as strip tests, with the project’s technical teams trained in their application. This transgene-detection method was chosen due to its accessibility and practicality in the field conditions of the projects and because it is an educational tool to provide guidance for family farmers in the region. Although the detection limits of these tests are known and well publicized by the companies responsible for their production and commercialization, they can be understood as indicators of the risk and degree of transgene flow in communities and could be confirmed and quantified by other methods with greater sensitivity to low positive levels, such as PCR or qPCR.

Considering the abovementioned elements, it is noted that the present study originated from an educational initiative structured in the urgency identified through the action of the Brazilian organized civil society. Thus, the limits of the methodology are known; however, due to the breadth of the analysis and given the alarming results that were found, there was an effort to disclose these results in the scientific literature, with the purpose of warning the scientific community about the subject and encouraging the conduction of other studies to enable a deeper understanding of the phenomenon identified. Moreover, considering the high costs required to carry out immunochromatographic or PCR tests, the observation of the results presented in this study becomes more important since it is rare to find studies with such amplitude. Farmers refer locally to transgene flow, either by cross-pollination or by seed mingling, as “contamination” to express their understanding of how foreign and novel genetic constructs protected by intellectual property rights might impact their seed varieties and seed-management systems.

The purpose of this study was to: (i) document transgene flow into landraces of maize conserved by family farmers in the SAB; (ii) demonstrate the efficiency of the rapid immunochromatographic test (strip test) as a tool for local transgene flow monitoring in landraces; and (iii) evaluate the implications of de-regulation and indiscriminate use of GMOs for the on-Farm conservation of genetic diversity in maize.

## 2. Results

The research data are presented highlighting the two periods in which they occurred because they represent two different harvests in subsequent years. Data were collected in different locations due to the actions of the projects (see Material and Methods). In this period, there were changes in immunochromatographic tests available in the market; thus, it is worth mentioning that this study has no intention to make comparisons between the cycles and that it would not be appropriate to address increased or decreased transgene flow but only to verify the phenomenon and its breadth across the years.

### 2.1. Transgene Flow Frequency

**Figure 1:**
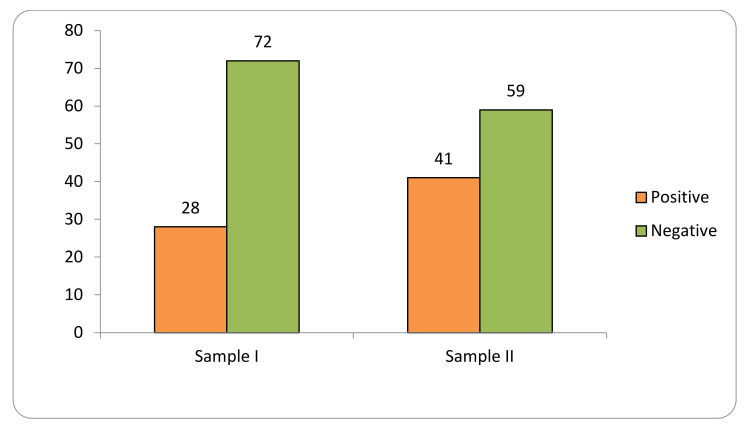
Transgene flow frequency into maize landraces, evaluated in two samples (Sample I—years 2018–2019; Sample II—years 2020–2021) in the Brazilian Semi-arid Region.

Transgene flow into maize landraces was detected in 34% of the 1098 collections evaluated in two samples at the SAB (2018–2019; 2020–2021) (Figure 1).

Sample I consisted of 576 samples, of which 28% tested positive for GM presence. Sample II consisted of 522 samples, of which 41% were positive.

### 2.2. Transgene Flow According to Seed-Management System 

**Figure 2:**
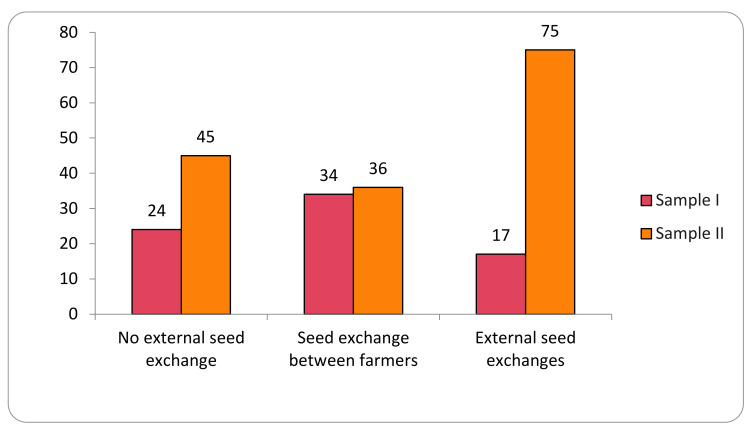
Transgene flow frequency into maize landraces, evaluated in two samples (Sample I—years 2018–2019; Sample II—years 2020–2021), considering the seed-management system.

The study identified 12 different origins of the varieties analyzed in the two samples. For analytical purposes, these origins are here grouped into three categories representing the main local seed-management system: (i) no external seed exchange; (ii) seed exchange between farmers; and (iii) external seed exchanges (Figure 2).

Seeds from the No External Seed Exchange group are cultivated and kept by the families in the family banks of their agroecosystems for periods of up to 250 years. In this category, 200 samples were evaluated in Sample I and 278 collections in Sample II, for a total of 478 collections. Collections from the family category represent 44% of the total collections evaluated.

The category Seed Exchange between Farmers comprised the origins that indicate (commercial or non-commercial) exchange relationships of seeds directly between farmers. These relationships included seeds from the same community (288 collections), other communities (103 collections), community seed houses or banks (63 collections), exchanges (38 collections), donations (20 collections), purchase among farmers (7 collections), seed fairs (14 collections), and international exchange (2 collections). This category represents 49% of the collections of the two samples evaluated, with 535 collections total.

The External Seed Exchange category comprised seeds not originated from another farmer or another community, that is, those seeds purchased in agricultural stores or producer/supplier fairs (24 collections) and seeds originated from public government policies (61 collections), making a total of 85 collection in the two samples. In the first sample evaluated, positive results were observed in 17% of the collections, and in the second sample, positive results were observed in 75% of the collections.

### 2.3. GM Proteins Detected

#### Grouping of GM Proteins by Mode of Action (Insect Resistance and Herbicide Tolerance)

**Figure 3:**
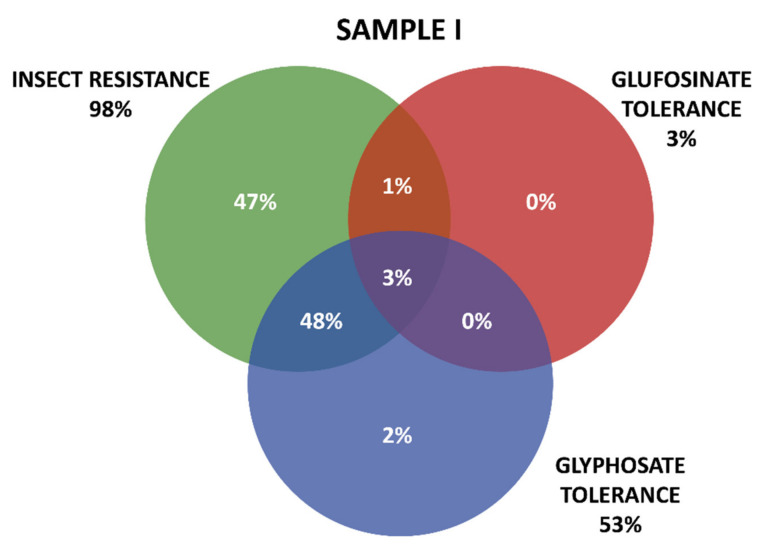
Detection and overlapping of GM proteins identified according to mode of action (insect resistance (IR) and tolerance to glufosinate ammonium (GAT) and glyphosate (GT) herbicides), Sample I.

**Figure 4:**
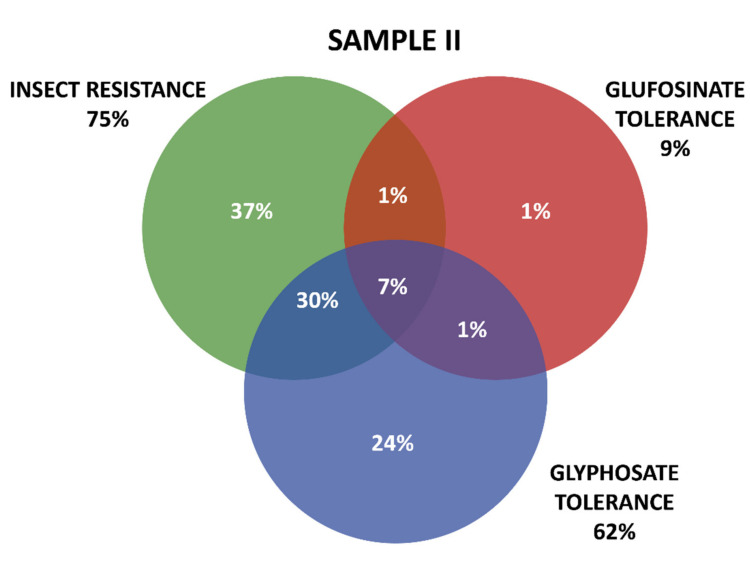
Detection and overlapping of GM proteins identified according to mode of action (insect resistance (IR) and tolerance to glufosinate ammonium (GAT) and glyphosate (GT) herbicides), Sample II.

Positive results were organized in three groups according the GM proteins mode of action. The first group contains the seven tested proteins that confer insect resistance (IR), and the other two groups comprise the proteins that provide tolerance to different active ingredients of herbicides (HT), that is, tolerances to glyphosate (GT) and to glufosinate-ammonium (GAT). The presence of GM proteins for IR was observed in the majority of the positive samples. Samples containing GT proteins appeared more frequently than those with GAT protein. Among IR proteins, those in the Cry group were predominant, consisting mainly of those that confer resistance to *Spodoptera frugiperda* and *Diabrotica speciosa*, important pests that affect maize crops in Brazil. In Sample I, 161 collections were positive, while in Sample II, positive results were observed in 213 collections, making up a total of 371 collections with at least one GM protein detected by the strip test. Further details on the detection of GM proteins in the samples can be seen in Table 1 and Figure 3 and Figure 4.

Figure 3 and Figure 4 show details of GM proteins frequency per mode of action and the occurrence of stacked events IR + TH.

For both samples, it was observed that the overlapping of the IR and GT groups is a situation that occurs with high frequency, while in Sample II, the resistance to insects alone (37%) and glyphosate (24%) also show high frequency (37%). 

## 3. Discussion

### 3.1. Unregulated Expansion of GMO Crops and Potential Effects of Transgene Flow into Maize Landraces Conserved by Family Farmers in the SAB 

GM plants currently cultivated on a commercial scale were predominantly designed for expression of traits that aim to facilitate agricultural operations in large areas [32]. In Brazil, herbicide tolerance and insect resistance (HT and IR) account for 94% of the events approved for commercialization [33]. The main GM crops are soy, maize, and cotton (95% of the total), which occupy more than 50 million hectares, according to industry data [34]. In the case of maize, until 2020, 53 GM varieties were authorized for commercial cultivation [35,36]. A survey for the 2019/2020 harvest indicates 26 maize cultivars with VT PRO3© technology and 25 with PowercoreTM Ultra© technology present in the seed market [35]. Proteins encoded from these GM events were widely found in the samples evaluated in this study, in agreement with the survey on the prevalence of these technologies.

As reported in Section 2.3, variable combinations and different proportions of these GM events were found in 34% of the 1098 samples analyzed. This unintentional pyramiding can give rise to the development of insect pests resistant to one or more toxins [7]. Zenner-de-Polanía (2021) [37] reported that the greatest concern regarding maize transgene flow is a potential damage to the diversity of existing breeds and the accumulation of transgenic DNA. Lonh et al. (2020) [38] observed degeneration of maize landraces in a study carried out in the state of Santa Catarina in the southern region of Brazil, where the transgenic protein Cry1AB was introduced in a landrace to monitor the evolution of gene expression and concentration as well as its effects on the mortality of insects *Helicoverpa armigera* and *Spodoptera littoralis*. It was concluded that unintentional crossing between landraces and transgenic varieties causes irreversible damage since gene transcription and insect control bioactivation is stable and reliable.

Farmers unaware of a likely transgene flow can select seeds with insecticidal function because the resistance given to insects, with the reduction of damage, will hide important and interesting characteristics that were previously selected for years by these farmers. Duncan and Werk [39] (2019) stated, on the other hand, that if a gene is inadvertently introgressed into landraces, the characteristics of the VT3Pro© protein are not expected to alter, for example, the plant’s response to biotic or abiotic stresses, except for protection against insects and herbicide tolerance traits. They also concluded that these genes must segregate like any endogenous gene, following Mendel’s laws. This statement is contested by studies that found that plant transformation is a complex event relying on both external and internal factors and that it is impossible to predict the exact way in which proteins involved in DNA replication interact with exogenous DNA and the genome of the recipient plant [40]. The expression of the new trait may be influenced by the position of transgene integration [41], a fact that is highly random even in controlled processes of plant modification [42]. The nature of the recipient genome, the referred transgenes, and the interactions between them might contribute to the non-Mendelian segregation of transgenes [43]. Maize landraces may be exposed to this situation.

The adverse effects of transgene insertion can also cause interactions between the physiological pathways of the plant, producing potentially harmful substances that can impact agricultural productivity [44]. Transgene flow can also change the phenotype or qualities of the contaminated plant [7], which are the attributes used by farmers to select seeds [45,46,47,48,49].

The transgene flow into maize and its consequences have the potential to profoundly impact food culture throughout Latin America. Across the region, different races were identified, reaching 64 in Mexico, considered its center of origin; 52 in Peru; 43 in Argentina; 40 in Bolivia; and 23 in Brazil [19]. This diversity is directly related to a wide variety of cultures, products, and uses. The ancient relationship between humans and maize is also shown in the different processing types that increase the nutritional value of the grain, facilitate grinding, and reduce the levels of antinutrients, such as phytate and mycotoxins [50]. Farmers in Yucatán, Mexico, cultivate a wide range of varieties with different agronomic characteristics in order to reduce risk of loss and increase their food and nutritional safety [50], a situation similar to that experienced by family farmers in the SAB.

In Paraguay, a study evaluated the presence of transgenic proteins in 18 samples of maize grain sold for the preparation of flour [51]. Based on PCR tests, five positive samples for the P-35S-CaMv promoter and five positive samples for the Terminator T-nos were identified. None of them presented the two sequences sought in the same sample, indicating that 10 out of 18 samples were positive. These results show that transgenic products are available in local markets in Paraguay without informing the consumers. This example demonstrates that the diversity of food crops is threatened by the genetic erosion of maize varieties, which involves agronomic, ecological, economic, and social factors [52].

In Brazil, a federal decree determines that every food or food ingredient that contains or is produced from GMOs with a presence above the 1% limit of the product must have this information on its packaging [53]. However, in relation to the commercialization of seeds, this information is not guaranteed. Many farmers who contributed to this study claim to have purchased maize seeds from the local market that were “stronger for the caterpillars” or “were able to withstand poison” (i.e., herbicides). They also informed that some seeds were purchased in fractions, that is, in amounts that are smaller than those formally commercialized (20 kg bags); therefore, they had no contact with the seed packaging containing company information, as determined by law. Thus, many of these farmers were unaware of whether or not these were GM seeds, the technology they are embedded with, or the risk they were taking of transgene flow into their seeds. The lack of information about the seeds acquired in the local market can explain the positive levels found in Samples I and II in the External Seed Exchange category, which were, respectively, 17% and 75%, with the significant increase justified by the greater demand for commercial seeds due to the reduction of drought.

Landraces are associated with safety and autonomy for cultivation since their seeds come from previous cultivations and are kept under the care of the subjects who cultivate them [54]. The collective action of farmers in SAB has enabled the prevention of reduction in the supply of maize varieties available for planting, as reported in Spain [55] and other regions of Brazil [56] as a result of the approval of transgenic varieties. Agapito-Tenfen et al. (2017) [11] analyzed two Mexican communities: community A cultivated both landraces and hybrid maize varieties, carrying out external seed exchanges and acquisitions in the external market, while community B cultivated only maize landraces and carried out only internal exchanges without external market purchases. In the first community, decisions are taken at the individual level and in the second, at the community level. In community A, GM presence was identified in 20% of the collected samples, while in B, no GM presence was identified. Most of the SAB communities are closer to the conditions and practices of community A both in relation to the proximity of other maize-cultivation areas as well as in decision making and the mixed cultivation of landrace seeds kept by the community and seeds of external origin, regarding which they have less control and knowledge. As pointed out in the results of this study, the External Seed Exchange category showed higher rates of transgene flow in both samples evaluated. In Sample I, the No External Seed Exchange category was the one with the lowest positive rate, a value that increased in Samples II, possibly due to a reduction in the drought and greater search for external seeds of unknown origin.

Zenner-de-Polanía (2021) [37] compared the benefits and harms of growing transgenic varieties in Latin America. Among the benefits, the author pointed out the reduction in the use of insecticides, with positive impacts on the fauna and decreased damages to the environment. The author himself pointed out that no statistical data were found in the literature on reduction in the use of synthetic products to control *S. frugiperda*, noting that the risks and limitations are much better documented than the benefits. Resistance to the use of the technology was predicted from the beginning and was observed around the world [18,57] so that monitoring of tolerance and resistance is mandatory in almost all countries that have authorized the use of Bt maize, whereas in Brazil, the developer of the GMO is authorized to request a waiver of monitoring, as discussed below in Section 3.3. As stated by Fatoretto et al. (2017) [58], *S. frugiperda* developed resistance to most transgenic Bt hybrids in a period of just 3 years in Brazil. Similarly, Argentina, Cuba, Colombia, and Puerto Rico also detected resistance to *S. frugiperda*.

Thus, in addition to the loss of genetic resources associated with the transgene flow into native seeds, further intensifying genetic erosion, the use of transgenics is a threat to the ecological diversity of insects and directly impacts both the productivity of transgenic maize plants and maize plant cultivation and productivity without the technology. Hilbeck and Schmidt (2006) [59] pointed out the need for research to assess the effect of Bt toxins on non-target organisms since studies have shown their lethal effect on these organisms and that the permanence of these toxins in the food chain is found in a concentration three times higher in insects than in the GM maize leaves they fed on. Some studies have observed effects on the development of the population of predators of insects directly affected by Bt corn due to the absence of prey, which may result in the non-survival of the predator, often is considered a beneficial insect. In another study, effects were found on prey predators directly affected by Bt corn, such as a longer duration of the larval stage, which can benefit predators of these species and reduce the number of individuals that reach adulthood [60]. In this study, lower weights in adulthood and lower fertility were also found, characteristics that are harmful to the population of this natural enemy. Furthermore, the presence of GMOs in the soil is already considered a source of pollution classified as a threat to soil biodiversity in the same category of intensive human exploitation, along with excessive soil disturbance, chemical fertilizers, and pesticides [61,62].

Drift of the herbicide widely sprayed on GM crops that reaches other areas where its use was not planned is also a source of already-documented damage. Eker et al. (2006) [63] reported reduced absorption of micronutrients and the reduced growth of shoots and root system of sunflower reached by underdosing of glyphosate-based products. A similar effect was observed in soybeans, according to Cakmak et al. (2009) [64], who identified a decrease in Ca, Mg, Fe, and Mn in soybeans affected by drift of glyphosate-based herbicides, an effect that also affected seed quality. Exposure of coffee plantations to unintentional underdosing of glyphosate can affect mycorrhizal activity and P absorption [65].

Zanatta et al. (2020) [44] showed that glyphosate-based herbicides trigger unwanted effects even in varieties genetically modified to resist this class of pesticides. The analysis revealed that glyphosate-based herbicides cause adverse effects on metabolism and central carbon flux, redox metabolism, photosynthesis, and the hormonal and defense response of plants. These data are relevant when taking into account that the increase in the area cultivated with GMOs in Brazil has corresponded to an increase in herbicide use, especially glyphosate-based ones [66]. Globally, glyphosate use has increased nearly 15-fold since 1996, with the advent of genetically modified crops with “Roundup Ready” technology, accounting for about 56% of glyphosate use worldwide [67]. There are controversial reports around the world about the benefits and harms of using glyphosate in social, economic, and environmental aspects as well as to the human health [68,69]. As shown above in Figure 3 and Figure 4, it was found that glyphosate-based herbicide tolerance genes are the most evident among the HT events identified in the positive samples. This situation is aggravated in Brazil by the flexibilization of socio-environmental legislation [70,71,72].

#### Decreased Drought and Increased Transgene Flow

**Figure 5:**
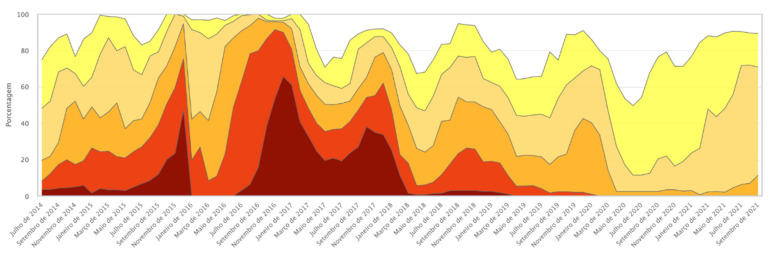
Drought tabular data history. Source: Drought Monitor. Available online: https://monitordesecas.ana.gov.br/dados-tabulares?tipo=8&area=5 (accessed on 10 November 2021).

The GM presence observed in both samples may be associated with the progressive increase in rainfall and the consequent reduction in the effects of the drought that began in 2012, bringing the average rainfall closer to levels considered normal for the region. The reduction in the intensity of drought in general provides better harvests; for maize, this means greater planted area and thus greater possibility of crossings and GM introgression. In 2018–2019, 576 immunochromatographic strip tests were performed, and the presence of at least one GM protein was detected in 28% of the maize landraces samples. In 2020–2021, a relative increase in GM presence was found, extending the number of maize landrace samples with at least one identified transgenic protein to 41%. The samples shown were owned by family farmers and are landrace varieties, that is, highly valuable in terms of genetic and cultural heritage, which indicates very significant positive results. Figure 5 shows the evolution of drought intensity in northeast Brazil between 2014 and 2021.

Table 2 shows the key for Figure 5.

### 3.2. Immunochromatographic Tests as a Tool for Local Transgene Flow Monitoring

Among the available methodologies for detecting transgenic proteins in plants, the strip test was the most feasible for this type of study. Although it only shows qualitative results, identifying the absence or presence of a particular protein, it plays a role in fast and efficient on-site traceability [73]. Other immunoassay methodologies were already proven efficient for the quantitative detection of GMOs [74]. It also has the advantage of being didactic as a participatory tool, a methodology used by the projects, allowing the tests to be carried out in community or family seed banks, along with the farmers who are seed keepers. For these reasons, strip tests have been adopted in different regions of the country to monitor transgene flow by local seed-management systems and agrobiodiversity conservation.

Digital PCR tests, real-time PCR, and sequencing (for identification of unauthorized GMOs) adopted by official inspection bodies have lower costs once the laboratories are installed and considering the equipment used. According to a survey carried out in this study, a sample analyzed with the strip test costs BRL 37.00 and in the laboratory with q-PCR the material costs BRL 8.00, excluding depreciation values for equipment, workforce, and infrastructure. However, equipment such as this is not easily accessible to family farmers and their organizations.

During the execution of the Semi-arid Agrobiodiversity Project, several training sessions were carried out with the executing team in the territories in order to deepen their theoretical knowledge on the subject, enabling them to better guide the seed keepers and reflect on territorial strategies to avoid transgene flow. An important moment of the team training was exclusively dedicated to methodological leveling, with the intent to carry out the strip tests with the farmers. The effectiveness of different methods to analyze the occurrence of transgenic events in maize seeds were discussed, either in the laboratory or through strip tests and others, and the protocols and field manual for carrying out immunochromatographic tests were introduced. Added to these opportunities to reflect on the transgene flow into maize varieties, other important activities took place culminating in the publication of technical communications on the multiplication of landraces and on participatory experimentation through trials of landraces [75,76].

### 3.3. Implications of GMO De-Regulation for on-Farm Conservation of Maize Genetic Diversity

Despite legally cultivating GMOs on a commercial scale since 2003, Brazil took time to adopt coexistence measures among transgenic varieties with conventional, agroecological, and organic crops [77,78,79]. The first commercially approved GM maize variety occurred in 2007 without the establishment of any specific isolation rules by the Brazilian biosafety authority, the National Biosafety Technical Commission (CTNBio). At the request of social organizations, the Justice Court suspended the decision until coexistence and monitoring rules were established. Additionally, in 2007, CTNBio defined rules aiming at “Establishing the minimum isolation distances to be observed between commercial crops of genetically modified corn and non-genetically modified corn crops, to allow the coexistence between the different production systems in the field” [80]. The standard determines that “To allow coexistence, the distance between a commercial crop of genetically modified corn and another of non-genetically modified corn, located in a neighboring area, must be equal to or greater than 100 (one hundred) meters or, alternatively, 20 (twenty) meters, provided that there is a border with at least 10 (ten) rows of conventional corn plants of size and vegetative cycle similar to genetically modified corn” (Ibid). The inadequacy of the standard is evident when compared to the directive of the Ministry of Agriculture, which requires a minimum distance of 400 m to isolate maize seed-production fields.

Studies carried out under different environmental conditions identified maize transgene flow at much greater distances. Hofmann et al. (2014) [81] identified in European countries that maize pollen reaches up to 1 km, and the dispersion decreases with distance. In Uruguay, maize GM pollen was identified 330 m from the source, with introgression of up to 1/40 seed in F1 [13]. GM gene flow in maize can occur through fertilization or mingling of grains [7]. Dyer et al. (2009) [82] found dispersion of GM seeds from the USA, identifying 3.1% of samples with Bt genes and 1.8% with RR genes. Additionally, by grain-blending processes, researchers identified 15% samples positive for RR and 10% for Bt in varieties grown by local farmers in South Africa [83]. In Venezuela, Díaz and Galindo (2014) [84] found samples of local maize with GM events that were not approved for cultivation in the country. In Japan, transgene flow was observed 800 m from the pollen source, with non-homogeneous rates varying with wind speed and incidence of solar radiation [85]. The recommendation to change the planting time could be an additional strategy to minimize transgene flow, but recent studies indicate that this measure affects the production and quality of seeds obtained outside the season [86].

Between 2007 and 2021, 53 GM maize varieties were commercially approved [36]. Random and varied combinations of these events are cultivated annually on about 40 million hectares in Brazil. The repeated and large-scale introduction of GM crops can increase their unintentional presence in the environment [7]. In 2020 [87] and again in 2021 [88], CTNBio changed its internal rules, allowing applicant companies to request exemption from the post-commercial release monitoring plan [89]. In 2015, with subsequent changes in 2020 [88] and 2021 [89], the national biosafety rules began to accept the automatic approval of GMOs with pyramid events whose individual events have been previously approved for commercial release by CTNBio, as requested by the applicant [90]. Controlling the unintentional and unplanned diffusion of GMOs still requires strategies that consider studies on the social and cultural dynamics of local seed systems and on how GMOs have entered and moved within rural communities [5,11].

## 4. Materials and Methods

### 4.1. Study Area and Sample Collection Points

**Figure 6:**
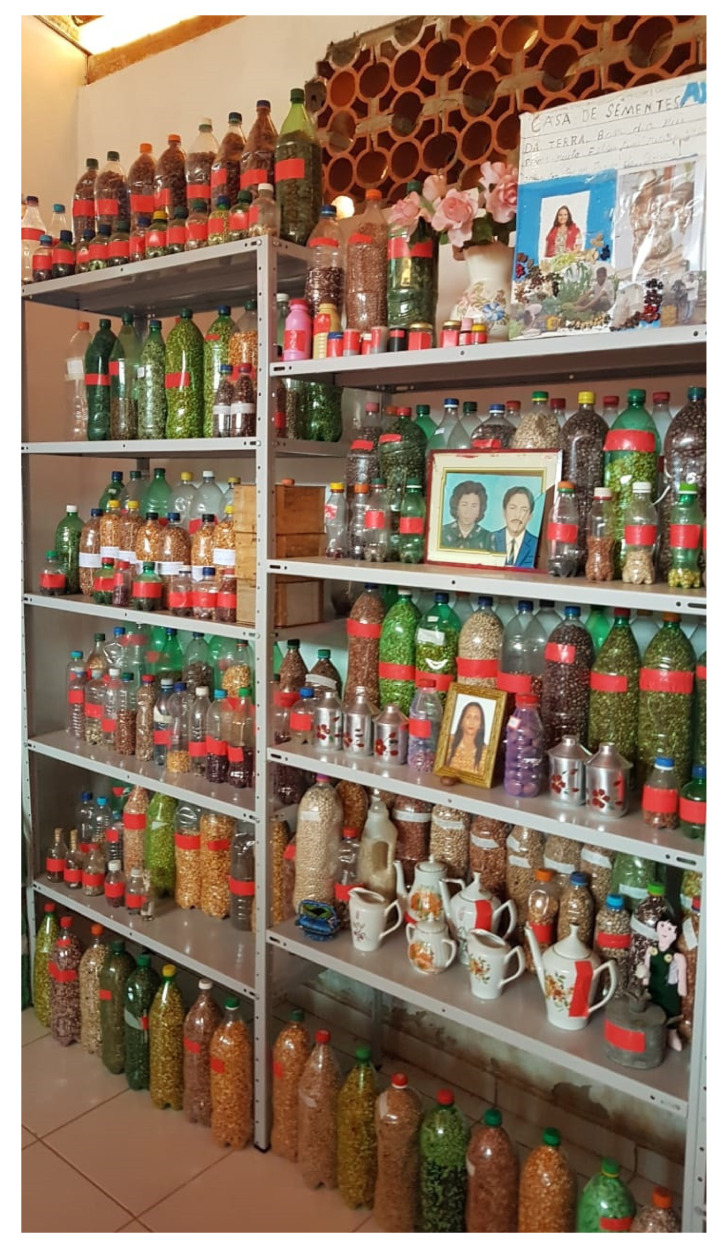
Community Seed Bank. Photo credit: Semi-arid Agrobiodiversity Management Program and Agrobiodiversity of the Semi-arid Project image bank, Bom Jesus da Lapa, Bahia, Brazil, October 2018.

This study analyzed 1098 samples of maize seeds preserved under on-Farm conditions by family farmers in family or community seed banks (Figure 6) residing in 138 municipalities in 9 states of the Brazilian Semi-arid Region, representing about 10% of the municipalities in the region (See supplementary material).

**Figure 7:**
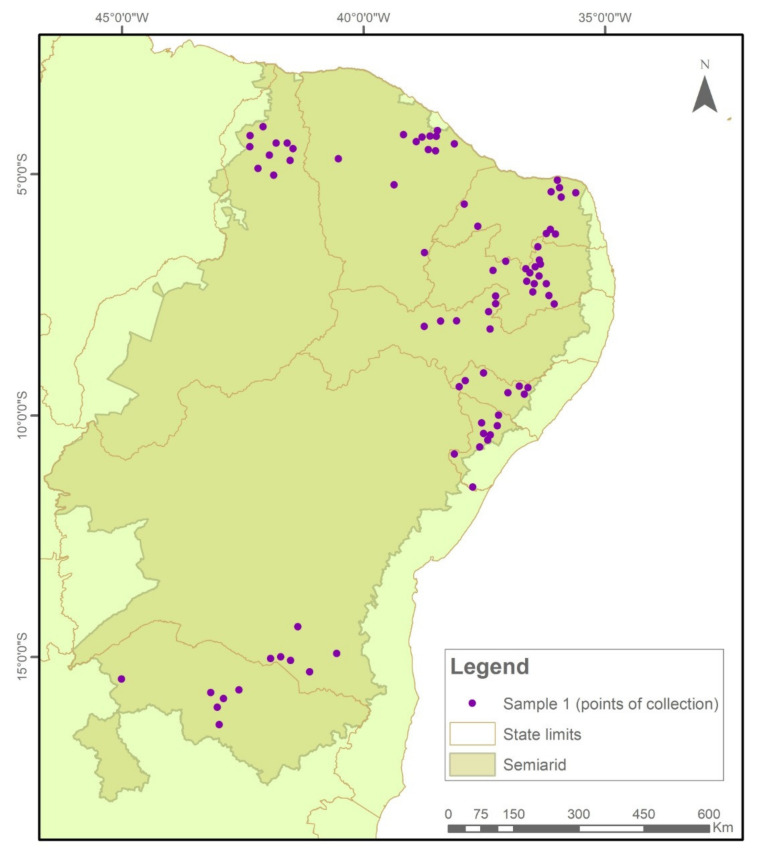
Indication of seed sample collection points in municipalities in the Brazilian Semi-arid Region, 2018–2019.

**Figure 8:**
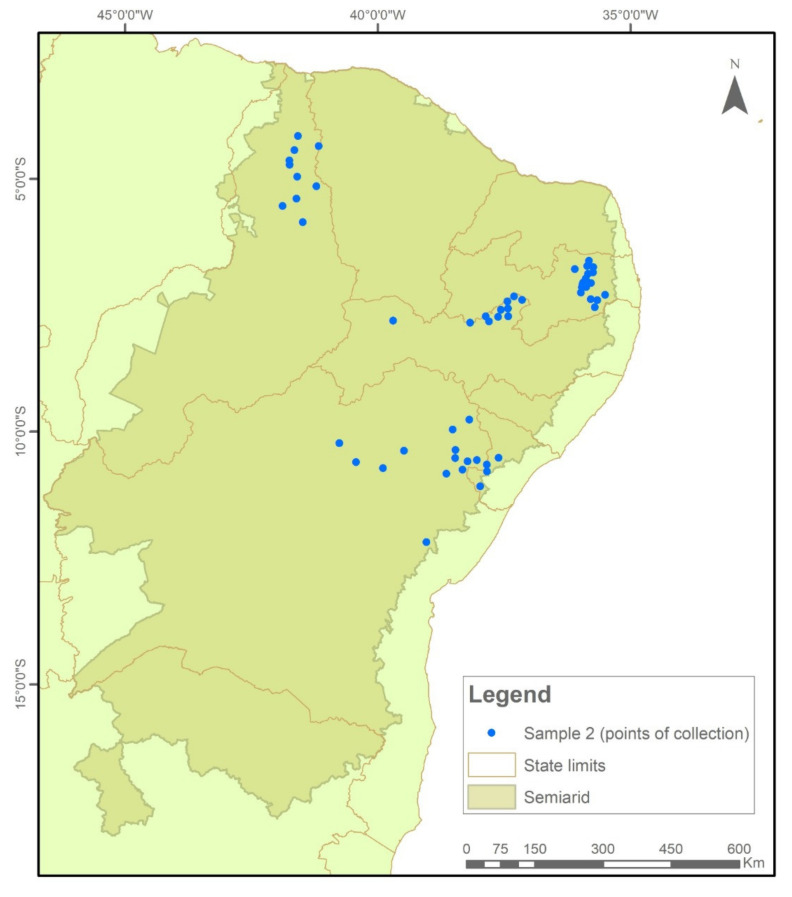
Indication of seed sample collection points in municipalities in the Brazilian Semi-arid Region, 2020–2021.

The samples were collected and tested in two temporal cycles. In Sample I (2018/2019), 576 collections were evaluated (Figure 7), while in Sample II (2020/2021), another 522 seed collections were evaluated (Figure 8).

### 4.2. Collections and Tests Performed

The tests were applied to maize seeds acknowledged as landraces, that is, varieties that have been under the management of farmers and guardians for at least 3 years. Generally speaking, holdings of family farmers in the SAB region are smaller if compared to the agribusiness in the region: the average is 29.2 ha, ranging from 12.2 ha in the state of Alagoas to 53.6 ha in the state of Minas Gerais [91], which hinders or prevents the creation of physical barriers to stop airborne GM pollen dispersion as well as to ensure the minimum mandatory distance for GM crops isolation. In the Brazilian Semi-arid Region, it is also important to consider that planting in different periods in order to prevent transgene flow is not a viable option due to the concentrated rainfall in short periods of the year.

**Figure 9:**
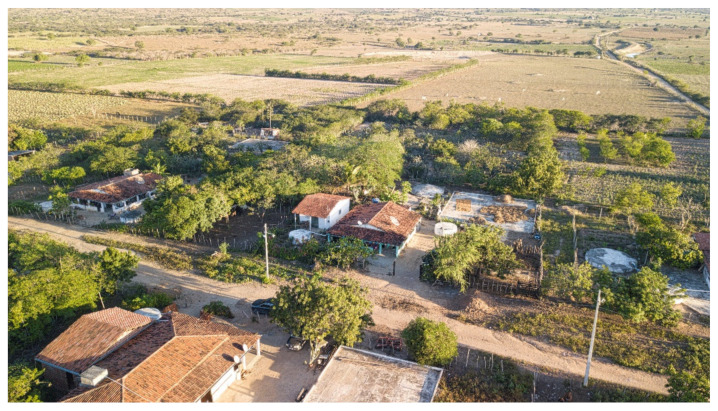
Aerial view of family farmers’ properties in the SAB, showing the great diversity of crops close to each other in small areas. Photo credit: Flavio Costa. Remígio, Paraíba, Brazil, May 2021.

It must also be taken into consideration that most of these areas are close or have easy access to urban centers, enabling access to seeds in the local market as well as allowing exchange with other family farmers. Therefore, these samples were collected in typical field conditions in the context of family farming in the Brazilian Semi-arid Region (Figure 9).

**Figure 10:**
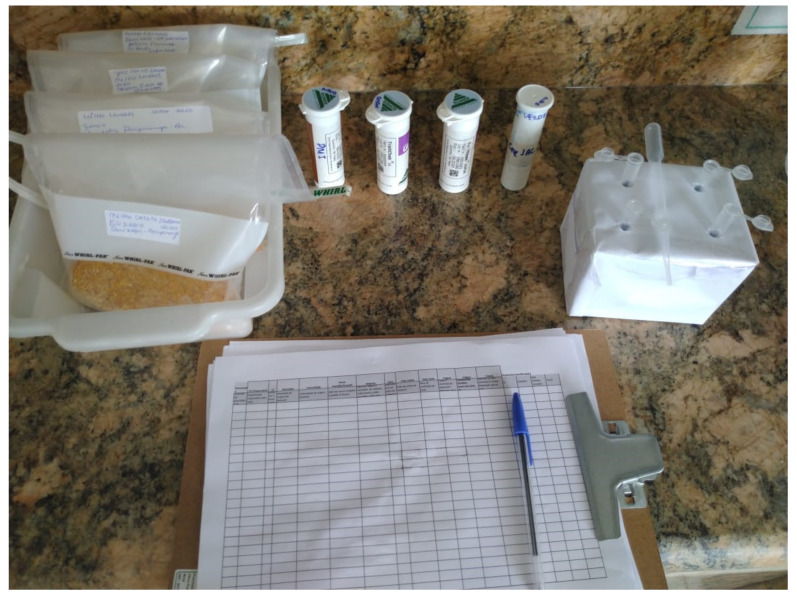
Identified samples of maize landraces seeds submitted to the strip test. Photo credit: Semi-arid Agrobiodiversity Management Program and Agrobiodiversity of the Semi-arid Project image bank. Cícero Dantas, Bahia, Brazil, February 2021.

At the time of the collection, sample seeds were properly identified with data regarding their origin. At least 10,000 grains were collected from each sample for detection by immunochromatographic test at 0.1% sensitivity (1/1000 seeds). The sample was manually homogenized in clean, disposable plastic bags. After homogenization, 250 g of sample grains were crushed. The remaining volume was stored for counterproof tests as needed (Figure 10).

Sample I was collected during the Semi-arid Agrobiodiversity Management Program—Seeds of the Semi-arid Region, in the harvest of 2018/2019. Collections were made in all states of the Brazilian Semi-arid Region, and tests were carried out for Liberty Link, Cry1F, Vip3A, CP4ESPS, Cry3Bb, Cry2AB, and Cry1Ab proteins.

Sample II was collected during the Inova Social Program, in the Semi-arid Agrobiodiversity Project, in 5 out of 10 states of the SAB region, involving the states of Bahia, Sergipe, Pernambuco, Piauí, and Paraíba. Tests were carried out for mEPSPS, Cry1A, Cry1Ab, Cry1Ac, Cry1A.105, Liberty Link, Cry3Bb, Cry1F, Cry34, and Cry2Ab proteins, some of them covered by stacking (stacking occurs when a GM protein is necessarily found associated to another protein; thus, it is also considered as identified although no specific testing was carried out for such protein. This procedure was adopted upon previous consulting with the technical team of the company in charge of the tests purchased since, in some cases, this was the only alternative because there were no tests available for all proteins authorized for commercialization in the country). An innovation for this sample was the use of the PMI test, which is capable of identifying the presence of a set of proteins, such as mCry3A, eCry3, and Vip3A, but without identifying which of them is present.

In both cases, the company that sold the tests was requested to provide a combination of tests that could offer the best coverage of the proteins authorized for commercialization in the country at the moment.

**Figure 11:**
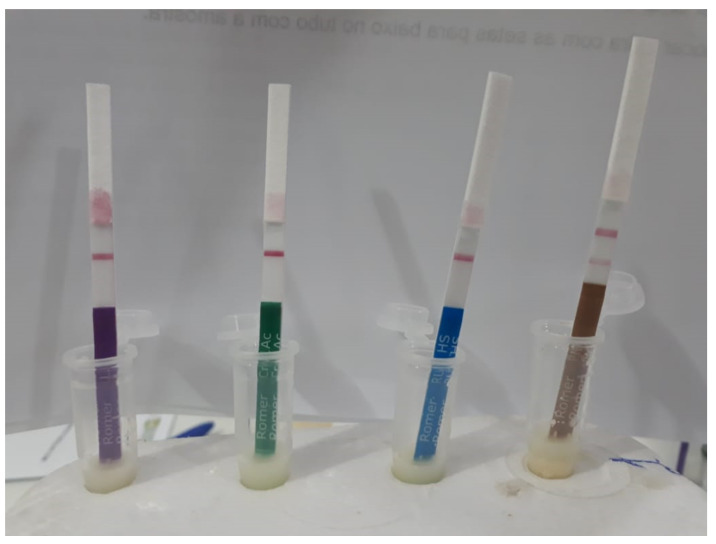
Example of test result carried out with farmers. Two lines indicate positive for GMO presence. Photo credit: Semi-arid Agrobiodiversity Management Program and Agrobiodiversity of the Semi-arid Project image bank. Embrapa Semiárido Seed Laboratory, Petrolina, Pernambuco, Brazil, January 2021.

The presence of GM proteins was determined by means of immunochromatographic tests (lateral flow strips or strip tests) (Figure 11). These tests identify one or more proteins that guarantee the screening of transgenic events commercially released for maize farming in Brazil, as shown in Table 3.

It is worth noting that in between Project 1 (2018–2019) and Project 2 (2020–2021), CTNBio granted commercial approvals for nine new GM maize varieties (Table 4). Strip test companies then updated marketed kits to cover these new events or combination of events (Table 3). The entry of these new varieties into the market prompted changes on the testing kits adopted by the projects. GM marketed strip tests consider that Cry1F-containing events appear stacked with CP4, Cry1Ab, PAT, and/or PMI (Vip3a and mCry3A), while Cry2Ab-containing events stack with CP4, Cry1Ab, Cry1a.105, PAT, and/or PMI. According to sellers, Cry1A tests scan for Cry1AB, Cry1AC, and Cry1a.105.

The samples were collected and tested in the presence of farmers by technical assistance and rural extension agents who received specific training to apply the protocol for carrying out immunochromatographic tests, based on the manufacturer’s technical recommendations, within the scope of the Semi-arid Seeds and Semi-arid Agrobiodiversity Projects.

**Figure 12:**
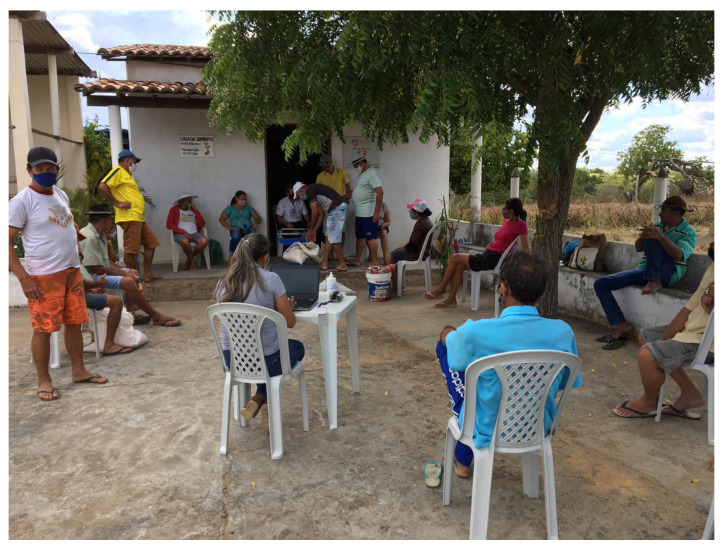
Capacity building meeting at a Community Seed Bank. Photo credit: Semi-arid Agrobiodiversity Management Program and Agrobiodiversity of the Semi-arid Project image bank. Fátima, Bahia, Brazil, February 2021.

In these projects, the immunochromatographic tests had an educational role, enabling farmers to view an abstract issue since it is impossible to identify transgene traits in a plant only through visual analysis (Figure 12). Thus, a few positive controls were made with transgenic seeds purchased in the market. These tests are not included in the material analyzed.

### 4.3. Data Analysis

Positive and negative tests were transformed into binary variables in a double-entry table (0.1), with 0 being negative and 1 being positive for the presence of transgenic protein.

Thus, the information was generated considering the parameters below:

(a) Frequency of positive seed lot of farmers’ landraces in immunochromatographic tests carried out in the two collection cycles 2018–2019 and 2020–2021;
(b) Frequency of positive results in seeds according to the seed-management system in the two collection cycles 2018–2019 and 2020–2021;
(c) Frequency of occurrence of the different proteins tested in seeds from varieties positive for transgenic events.

## 5. Conclusions

Previous studies have confirmed the presence of transgenes in landraces without, however, discussing which strategies farmers have been adopting to monitor the risk of transgene flow considering the role they play in the on-Farm conservation of maize genetic diversity. Here, we present the unprecedented and scaled-up effort led by civil society organizations to monitor transgene flow into maize landraces. The results indicate ubiquitous presence of GM events in maize landraces conserved by family farmers in the SAB. In total, 1098 samples were evaluated in a participatory way by means of strip tests over 4 years, revealing positive results for the presence of GMOs in 34% of the samples, with up to seven different GM events in the same sample. Among the evaluated seed-management systems, higher levels of GM presence were found in the External Seed Exchanges category. In Cycle I of evaluation, the Seed Exchange between Farmers category presented a higher rate of positive samples than the No External Seed Exchange category. In Cycle II, the positive rate in the No External Seed Exchange category surpassed that of the Seed Exchange between Farmers category. Testing kits specifications changed from one project to the other, aiming to follow the entry of new GM maize varieties into the country seed market as well as to ensure the confidence in the tool’s tracking capacity. The occurrence of transgene flow into maize landraces may be even greater given that our sample base is limited to the working area of organizations linked to the ASA and to the projects in force in both periods. Nevertheless, this limitation reinforces the relevance of the results found and the challenges identified for on-Farm conservation of maize diversity.

The adoption of strip tests proved to be efficient for the participatory transgene-flow monitoring. In addition to the reliability of the results, the methodology has shown characteristics, such as easy application and quick access to the results, that are educational when adopted in the context of interinstitutional projects aimed at agrobiodiversity conservation. The high costs of testing are a bottleneck for the maintenance and expansion of this process. It is not yet clear how seed crops derived from New Breeding Technologies, such as gene editing, will be traced at the field level and whether testing tools will be available for participatory monitoring of these new seeds. There is a need for projects and policies supporting the civil society, research, and farmers’ organizations, enabling long-term transgene flow monitoring.

Commercial approval of GM varieties in Brazil occurs at a faster pace than the country’s ability to adopt effective measures to protect landraces and farmers’ seed-management systems. This discrepancy is aggravated by the accelerated trend of the regulatory body to promote measures to make the biosafety rules more flexible, such as the automatic approval of stacked events and the possibility of exempting the GMO developer from post-commercial release monitoring. Based on the results found, we suggest there is a direct relationship between the flexibility of commercial releases of GM maize and the insufficiency of national rules for controlling gene flow mediated by GM pollen or propagules with the increasing transgene flow into landraces in the studied region. Additional studies can assess this situation in other regions of the country. Studies are needed to assess the medium- and long-term effects of the presence of GMOs into the genetic diversity of varieties cultivated by farmers as well as on the sociocultural and economic systems responsible for the dynamic conservation of landrace seeds. CTNBio’s Normative Resolutions on isolation of GM maize, post-commercial-release monitoring, and approval of pyramid events should be revised. Transgene flow can be reduced by approving and enforcing more effective coexistence rules that consider maize landraces crop areas also as seed-producing areas added to full disclosure of commercial seeds origin. Effective measures are needed to confine GM seeds in the areas and agricultural systems for which they were designed, thus preventing the social sectors responsible for on-Farm conservation from assuming the burden of monitoring actions and the threat of losing their rights and their seeds.

## Figures and Tables

**Table 1:** Detection of protein by mode of action (insect resistance (IR) and tolerance to glyphosate (GT) and glufosinate-ammonium (GAT) herbicides) in Sample I (2018–2019) and Sample II (2020–2021).

Mode of Action	Tested Protein	Sample I	Sample II
Total positive collections		161	213
IR	VIP3A, Bt-Cry1A, Bt-Cry3Bb, Bt-Cry1F, Bt-Cry2Ab, mCry3A, Cry1Ac	158	159
GAT	PAT	5	19
GT	CP4 EPSPS	84	131
**Mode of Action**	**Tested Protein**	**Total Positive Samples**	
IR	VIP3A, Bt-Cry1A, Bt-Cry3Bb, Bt-Cry1F, Bt-Cry2Ab, mCry3A, Cry1Ac	317	
GAT	PAT	24	
GT	CP4 EPSPS	215	

**Table 2:** Drought stages or categories that define the drought intensity on the Monitor map. Source: Drought Monitor, Adapted from the National Drought Mitigation Center, Lincoln, Nebraska, U.S. Available online: https://monitordesecas.ana.gov.br/perguntas-frequentes (accessed on 10 November 2021).

Category	Percentile	Description	Potential Impacts
S0	30%	Weak Drought	Entering drought: short-term heat wave decreasing planting, crop growth, or grazing. Coming out of drought: some prolonged water deficits, pastures or crops not fully recovered.
S1	20%	Moderate Drought	Some damage to crops, pastures, streams, reservoirs, or wells with low levels, some developing or imminent water shortages; voluntary restrictions on water use requested.
S2	10%	Severe drought	Potential crop or pasture losses; regular water shortages; water restrictions imposed.
S3	5%	Extreme drought	Large crop/pasture losses; widespread water scarcity or restrictions
S4	2%	Exceptional Drought	Exceptional and widespread crop/pasture losses; scarcity of water in reservoirs, streams, and water wells, creating emergency situations.

**Table 3:** Tests, proteins/trace, and detection limits used in each of the evaluation samples.

Company	Product	Event (Trait)	Detection Limit (%)
2018/2019			
Romer Labs	AgraStrip© Triple Trait Bulk Grain	CP4 EPSPS	0.1
		Bt-Cry1A (Bt-Cry1Ab, Bt-Cry1Ac, Bt-Cry1A.105), Bt-Cry3Bb	9.5
	AgraStrip© Cry1F Bulk Grain (water extract)	Bt-Cry1F	0.9
	AgraStrip© Cry2Ab Bulk Grain	Bt-Cry2Ab	0.9
	AgraStrip© VIP Bulk Grain	VIP3A	0.33
	AgraStrip© LL Bulk Grain	PAT	0.9
2020/2021			
Romer Labs	AgraStrip© PMI Bulk Grain	PMI (Vip3A, mCry3A)	0.5
	AgraStrip© RUR-HS Bulk Grain	CP4 EPSPS	0.125
	AgraStrip© Cry1Ac Bulk Grain	Cry1Ac	0.9
	AgraStrip© LL Bulk Grain	PAT	0.9

Source: Romer Labs. Available online: https://www.romerlabs.com/pt/produtos/kits-de-analises/ogm/ (accessed on 1 November 2021).

**Table 4:** GM maize commercial approvals in Brazil from 2018 to 2021.

Year	Unique Identifier	Event (Trait)	Protein
2018	MON-89Ø34-3 x DAS-Ø15Ø7-1 x MON-ØØ6Ø3-6 x SYN-IR162-4 x DAS-4Ø278-9	MON 89034 x TC1507 x MIR162 x NK603 x DAS-40278-9	Cry1A105, Cry2Ab2, Cry1F and Vip3Aa20, PAT, CP4 epsps and AAD-1
	SYN-ØØØ98-3	MZIR098 (food/feed)	mCry3A/eCry3.1Ab
2019	MON-87427-7 x MON-89Ø34-3 x DAS-Ø15Ø7-1 x MON-87411-9 x DAS-59122-7 x DAS-4Ø278-9	MON-87427-7 x MON-89034-3 x DAS-01507-1 x MON-87411-9 x DAS-59122-7 x DAS-40278-9	CP4 epsps (aroA:CP4), Cry2Ab2, Cry1A.105, Cry1F, PAT, Cry34Ab1, Cry35Ab1, Cry3Bb
	MON-87427-7 x MON87419-8 x MON-ØØ6Ø3-6	MON 87427 × MON 87419 × NK603	CP4 EPSPS, DMO and PAT
	MON-87427-7 x MON-89Ø34-3 x SYN-IR162-4 x MON-ØØ6Ø3-6	MON 87427 x MON 89034 x MIR162 x NK603 (and subcombinations)	CP4 EPSPS, Cry1 A.1 05, Cry2Ab2 and Vip3Aa
2020	MON-ØØ6Ø3-6 x ACS-ZMØØ3-2 X DAS-4Ø278-9	NK603 x T25 x DAS-40278	CP4 EPSPS, PAT, AAD1
	MON-89Ø34-3 x DAS-Ø15Ø7-1 x MON-ØØ6Ø3-6 x SYN-IR162-4 x DAS-4Ø278-9	MON-89034-3 x DAS-01507-1 x SYN-IR162-4 x MON-00630-6 x DAS40278-9 (and subcombinations)	Cry1A.105 e Cry2Ab2, Cry1F, PAT, VIP3Aa20, CP4 EPSPS, AAD-1
	MON-95379-3	MON 95379	Cry1Da_7, Cry1B.868, Cry1Be, Cry1Ca, Cry1Ab
2021	DP-ØØ4114-3	DP4114-3	Cry1F, Cry34Ab1 and Cry35Ab1, PAT

Source: CTNBio: https://ctnbio.mctic.gov.br/liberacao-comercial?#/liberacao-comercial/consultar-processo (accessed on 19 December 2021).

## Data Availability

The data presented in this study are available in supplementary material.

## Supplementary Materials

The following are available online at https://www.mdpi.com/article/10.3390/plants11050603/s1. File: Study Area and Sample Collection Points.

## References

[1] Ellstrand N.C. (2001). When Transgenes Wander, Should We Worry?. Plant Physiol..

[2] Gepts P., Papa R. (2003). Possible effects of (trans)gene flow from crops on the genetic diversity from landraces and wild relatives. Environ. Biosaf. Res..

[3] Altieri M.A. (2005). The Myth of Coexistence: Why Transgenic Crops Are Not Compatible With Agroecologically Based Systems of Production. Bull. Sci. Technol. Soc..

[4] McAfee K. (2003). Corn Culture and Dangerous DNA: Real and Imagined Consequences of Maize Transgene Flow in Oaxaca. J. Lat. Am. Geogr..

[5] Binimelis R. (2008). Coexistence of Plants and Coexistence of Farmers: Is an Individual Choice Possible?. J. Agric. Environ. Ethic.

[6] Levidow L., Boschert K. (2008). Coexistence or contradiction? GM crops versus alternative agricultures in Europe. Geoforum.

[7] Heinemann J.A. (2007). Typology of the Effects of (Trans)Gene Flow on the Conservation and Sustainable Use of Genetic Resources.

[8] Vázquez-Barrios V., Boege K., Sosa-Fuentes T.G., Rojas P., Wegier A. (2021). Ongoing ecological and evolutionary consequences by the presence of transgenes in a wild cotton population. Sci. Rep..

[9] FAO (2009). International Treaty on Plant Genetic Resources for Food and Agriculture. https://www.fao.org/plant-treaty/en/.

[10] De Schutter O. (2009). Seed Policies and the Right to Food: Enhancing Agrobiodiversity, Encouraging Innovation Presented at the 64th Session of the UN General Assembly.

[11] Agapito-Tenfen S., Lopez F.R., Mallah N., Abou-Slemayne G., Trtikova M., Nodari R., Wickson F. (2017). Transgene flow in Mexican maize revisited: Socio-biological analysis across two contrasting farmer communities and seed management systems. Ecol. Evol..

[12] Quist D., Chapela I.H. (2001). Transgenic DNA introgressed into traditional maize landraces in Oaxaca, Mexico. Nature.

[13] Galeano P., Debat C.M., Ruibal F., Fraguas L.F., Galván G.A. (2010). Cross-fertilization between genetically modified and non-genetically modified maize crops in Uruguay. Environ. Biosaf. Res..

[14] Piñeyro-Nelson A., Van Heerwaarden J., Perales H.R., Serratos-Hernández J.A., Rangel A., Hufford M.B., Gepts P., Garay-Arroyo A., Rivera-Bustamante R., Álvarez-Buylla E.R. (2009). Transgenes in Mexican maize: Molecular evidence and methodological considerations for GMO detection in landrace populations. Mol. Ecol..

[15] Santa-Maria M.C., Lajo-Morgan G., Guardia L. (2014). Adventitious presence of transgenic events in the maize supply chain in Peru: A case study. Food Control.

[16] Knispel A.L., McLachlan S.M., Van Acker R.C., Friesen L.F. (2008). Gene Flow and Multiple Herbicide Resistance in Escaped Canola Populations. Weed Sci..

[17] Schafer M.G., Ross A.A., Londo J., Burdick C.A., Lee E.H., Travers S.E., Van De Water P.K., Sagers C.L. (2011). The Establishment of Genetically Engineered Canola Populations in the U.S. PLoS ONE.

[18] Ferment G., Melgarejo L., Fernandes G.B., Ferraz J.M.G. (2017). Transgenic Crops–Hazards and Uncertainties: More than 750 Studies Disregarded by the GMOs Regulatory Bodies.

[19] Silva N.C.d.A., Costa F.M., Vidal R., Veasey E.A. (2020). Maíces de Las Tierras Bajas de América Del Sur y Conservación de La Agrobiodiversidad En Brasil y Uruguay.

[20] Kistler L., Maezumi S.Y., de Souza J.G., Przelomska N.A.S., Costa F.M., Smith O., Loiselle H., Ramos-Madrigal J., Wales N., Ribeiro E.R. (2018). Multiproxy evidence highlights a complex evolutionary legacy of maize in South America. Science.

[21] Costa F.M., Silva N.C.D.A., Vidal R., Clement C.R., Alves R.P., Bianchini P.C., Haverroth M., Freitas F.D.O., Veasey E.A. (2021). Entrelaçado, a rare maize race conserved in Southwestern Amazonia. Genet. Resour. Crop Evol..

[22] Carvalho V.P., Ruas C.F., Ferreira J.M., Moreira R.M., Ruas P.M. (2004). Genetic diversity among maize (*Zea mays* L.) landraces assessed by RAPD markers. Genet. Mol. Biol..

[23] Silva N.C.D.A., Vidal R., Costa F.M., Veasey E.A. (2020). Clasificación de Las Razas de Maíz de Brasil y Uruguay: Enfoque Metodológico y Principales Resultados. Maíces de las Tierras Bajas de América del Sur y Conservación de la Agrobiodiversidad en Brasil y Uruguay.

[24] Costa F.M., Silva N.C.D.A., Vidal R., Veasey E.A. (2020). Micro-Centros de Diversidade Genética Do Milho Nas Terras Baixas Da América Do Sul. Milhos das Terras Baixas da América do Sul e Conservação da Agrobiodiversidade no Brasil e no Uruguai.

[25] Silva N.C.D.A., Vidal R., Ogliari J.B. (2017). New popcorn races in a diversity microcenter of Zea mays L. in the Far West of Santa Catarina, Southern Brazil. Genet. Resour. Crop Evol..

[26] Costa F.M., Silva N.C.D.A., Ogliari J.B. (2017). Maize diversity in southern Brazil: Indication of a microcenter of *Zea mays* L.. Genet. Resour. Crop Evol..

[27] Silva N.C.D.A., Costa F.M., Vidal R., Veasey E.A. (2020). Microcentros de Diversidad Genética Del Maíz En Las Tierras Bajas de América Del Sur. Maíces de las Tierras Bajas de América del Sur y Conservación de la Agrobiodiversidad en Brasil y Uruguay.

[28] Fiore M.C., Raimondo F.M., Mercati F., DiGangi I., Sunseri F., Scialabba A. (2020). Preserving Biodiversity in Marginal Rural Areas: Assessment of Morphological and Genetic Variability of a Sicilian Common Bean Germplasm Collection. Plants.

[29] Bellon M.R., Hodson D., Hellin J. (2011). Assessing the vulnerability of traditional maize seed systems in Mexico to climate change. Proc. Natl. Acad. Sci. USA.

[30] Galluzzi G., Seyoum A., Halewood M., Noriega I.L., Welch E.W. (2020). The Role of Genetic Resources in Breeding for Climate Change: The Case of Public Breeding Programmes in Eighteen Developing Countries. Plants.

[31] Maronhas M.E.S., Pinilla N.N., Lira J.L. (2020). Caminos Del Semiárido Brasileño Para La Gestión de La Agrobiodiversidad. Biodiversidad LA. https://www.biodiversidadla.org/Documentos/Caminos-del-Semiarido-brasileno-para-la-gestion-de-la-agrobiodiversidad.

[32] Fischer K. (2016). Why new crop technology is not scale-neutral—A critique of the expectations for a crop-based African Green Revolution. Res. Policy.

[33] CTNBio (2021). Resumo Geral de Plantas Geneticamente Modificadas Aprovadas Para Comercialização. Ministério da Ciência, Tecnologia, Inovações e Comunicação: Brasília, Brazil.

[34] ISAAA (2017). Global Status of Commercialized Biotech/GM Crops in 2017: Biotech Crop Adoption Surges as Economic Benefits Accumate in 22 Years.

[35] Pereira Filho I.A., Borghi E. (2020). Sementes de Milho: Nova Safra, Novas Cultivares e Continua a Dominância Dos Transgênicos.

[36] CTNBio Liberações Comerciais-Milho. Ministério da Ciência, Tecnologia, Inovações e Comunicação: Brasília, Brazil 2020.

[37] Zenner de Polania I. (2021). Transgenic Bt Maize in South-and Central America: The Pros and Cons. Rev. Colomb. Cienc. Hortícolas.

[38] Lohn A.F., Trtikova M., Chapela I., Berg J.V.D., Du Plessis H., Hilbeck A. (2020). Transgene behavior in *Zea mays* L. crosses across different genetic backgrounds: Segregation patterns, cry1Ab transgene expression, insecticidal protein concentration and bioactivity against insect pests. PLoS ONE.

[39] Duncan B., Leyva-Guerrero E., Werk T., Stojšin D., Baltazar B.M., García-Lara S., Zavala-López M., De La Fuente-Martínez J.M., Meng C. (2019). Assessment of potential impacts associated with gene flow from transgenic hybrids to Mexican maize landraces. Transgenic Res..

[40] Kohli A. (2003). Transgene integration, organization and interaction in plants. Plant Mol. Biol..

[41] Kumar S., Fladung M. (2001). Gene stability in transgenic aspen (Populus). II. Molecular characterization of variable expression of transgene in wild and hybrid aspen. Planta.

[42] Latham J.R., Wilson A.K., Steinbrecher R.A. (2006). The Mutational Consequences of Plant Transformation. J. Biomed. Biotechnol..

[43] Yin Z., Plader W., Malepszy S. (2004). Transgene inheritance in plants. J. Appl. Genet..

[44] Zanatta C.B., Benevenuto R.F., Nodari R.O., Agapito-Tenfen S.Z. (2020). Stacked genetically modified soybean harboring herbicide resistance and insecticide rCry1Ac shows strong defense and redox homeostasis disturbance after glyphosate-based herbicide application. Environ. Sci. Eur..

[45] Louette D., Smale M. (2000). Farmers’ seed selection practices and traditional maize varieties in Cuzalapa, Mexico. Euphytica.

[46] Louette D., Charrier A., Berthaud J. (1997). In Situ conservation of maize in Mexico: Genetic diversity and Maize seed management in a traditional community. Econ. Bot..

[47] Bellon M., Brush S.B. (1994). Keepers of maize in Chiapas, Mexico. Econ. Bot..

[48] Louette D. (2000). Traditional Management of Seed and Genetic Diversity: What Is a Landrace?. Genes in the field on-farm conservation of crop diversity.

[49] Hugo P.R., Brush S.B., Qualset C.O. (2003). Dynamic Management of Maize Landraces in Central Mexico. Econ. Bot..

[50] Guzzon F., Rios L.W.A., Cepeda G.M.C., Polo M.C., Cabrera A.C., Figueroa J.M., Hoyos A.E.M., Calvo T.W.J., Molnar T.L., León L.A.N. (2021). Conservation and Use of Latin American Maize Diversity: Pillar of Nutrition Security and Cultural Heritage of Humanity. Agronomy.

[51] Melgarejo M.E.D., Estigarribia P.E.P., Iehisa J.M., Arrua J.M.M., Martínez C.C.C., Arrua A.A. (2020). Contamination of corn grain for human consumption with transgenic sequences in Paraguay. Food Sci. Technol..

[52] Bellon M. (1991). The ethnoecology of maize variety management: A case study from Mexico. Hum. Ecol..

[53] (2003). Brasil Decreto, N. 680, de 24 de Abril de 2003.

[54] Lacey H. (2000). Seeds and the Knowledge They Embody. Peace Rev..

[55] Hilbeck A., Lebrecht T., Vogel R., Heinemann J.A., Binimelis R. (2013). Farmer’s choice of seeds in four EU countries under different levels of GM crop adoption. Environ. Sci. Eur..

[56] Capellesso A.J., Cazella A.A., Filho A.L.S., Farley J., Martins D.A. (2016). Economic and environmental impacts of production intensification in agriculture: Comparing transgenic, conventional, and agroecological maize crops. Agroecol. Sustain. Food Syst..

[57] Hilbeck A., Binimelis R., Defarge N., Steinbrecher R., Székács A., Wickson F., Antoniou M., Bereano P.L., Clark E.A., Hansen M. (2015). No scientific consensus on GMO safety. Environ. Sci. Eur..

[58] Fatoretto J.C., Michel A.P., Silva-Filho M., Silva N. (2017). Adaptive Potential of Fall Armyworm (Lepidoptera: Noctuidae) Limits Bt Trait Durability in Brazil. J. Integr. Pest Manag..

[59] Hilbeck A., Schmidt J.E.U. (2006). Another View on Bt Proteins–How Specific Are They and What Else Might They Do?. Biopestic. Int..

[60] Curis M.C., Bertolaccini M.A.T.I. (2013). Influência de presas criadas sobre maíces Bt sobre parámetros biológicos de Eriopis connexa (Coleoptera: Coccinellidae). Rev. Ciênc. Agrár..

[61] Tibbett M., Fraser T.D., Duddigan S. (2020). Identifying potential threats to soil biodiversity. PeerJ.

[62] Aksoy E., Louwagie G., Gardi C., Gregor M., Schröder C., Löhnertz M. (2017). Assessing soil biodiversity potentials in Europe. Sci. Total Environ..

[63] Eker S., Ozturk L., Yazici A., Erenoglu B., Romheld V., Cakmak I. (2006). Foliar-Applied Glyphosate Substantially Reduced Uptake and Transport of Iron and Manganese in Sunflower (*Helianthus annuus* L.) Plants. J. Agric. Food Chem..

[64] Cakmak I., Yazici A., Tutus Y., Ozturk L. (2009). Glyphosate reduced seed and leaf concentrations of calcium, manganese, magnesium, and iron in non-glyphosate resistant soybean. Eur. J. Agron..

[65] Carvalho F.P., França A.C., Franco M.H.R., Avelar M., Moreira S.D., Alecrim A.O., Dos Santos J.B. (2014). Sensibilidade de plantas de café micorrizadas à herbicidas. Rev. Bras. de Herbic..

[66] De Almeida V.E.S., Friedrich K., Tygel A.F., Melgarejo L., Carneiro F.F. (2017). Use of genetically modified crops and pesticides in Brazil: Growing hazards. Ciência Saúde Coletiva.

[67] Benbrook C.M. (2016). Trends in glyphosate herbicide use in the United States and globally. Environ. Sci. Eur..

[68] Binimelis R., Pengue W., Monterroso I. (2009). “Transgenic treadmill”: Responses to the emergence and spread of glyphosate-resistant johnsongrass in Argentina. Geoforum.

[69] Pengue W.A. (2005). Transgenic Crops in Argentina: The Ecological and Social Debt. Bull. Sci. Technol. Soc..

[70] Melgarejo L., Issberner L.-R., Léna P. (2018). Biosafety Regulations and Practices and Consequences in Brazil: Who Wants to Hide the Problems?. Brazil in the Anthropocene: Conflicts between Predatory Development and Environmental Policies.

[71] Souza M.M.O., Gurgel A.d.M., Fernandes G.B., Melgarejo L., Bittencourt N.A., Friedrich K. (2020). Agrotóxicos e Transgênicos: Retrocessos Socioambientais e Avanços Conservadores No Governo Bolsonaro. Rev. Anpege.

[72] Petry C., Calliari M.R.T., Melgarejo L., Fernandes G.B., Bittencourt N.A., Souza M.M.O., Mulinari J., Reichert Júnior R., Francisco W., Mossi A.J., Petry C., Reichert F.W. (2020). Food in Security, Agrochemicals, New Biotechnologies and Democracy. Agroecology: Insights, Experiences and Perspectives.

[73] Lipton C.R., Dautlick J.X., Grothaus G.D., Hunst P.L., Magin K.M., Mihaliak C.A., Rubio F.M., Stave J.W. (2000). Guidelines for the Validation and Use of Immunoassays for Determination of Introduced Proteins in Biotechnology Enhanced Crops and Derived Food Ingredients. Food Agric. Immunol..

[74] Stave J.W. (1999). Detection of new or modified proteins in novel foods derived from GMO–future needs. Food Control.

[75] Curado F.F., Santos A.D.S., Fagundes R.d.C., Silva A.C.d.L., Bianchini P.C. (2020). Manejo Comunitário Da Agrobiodiversidade: Produção Agroecológica de Sementes de Variedades Crioulas Por Agricultores Familiares.

[76] Dos Santos A.D., Curado F.F., Silva A.C.d.L., Bianchini P.C., Fagundes R.d.C. (2020). Manual de Instalação de Ensaios Participativos Para Comparação de Variedades Crioulas.

[77] Ferment G., Zanoni M., Brack P., Kageyama P., Nodari R.O. (2009). Coexistência: O Caso Do Milho: Proposta de Revisão Da Resolução Normativa N 4 Da CTNBio.

[78] Fernandes G.B., Fernandes G.B., Ferment G., Avanci J. (2010). Seminário Sobre Proteção Da Agrobiodiversidade e Direito Dos Agricultores: Propostas Para Enfrentar a Contaminação Transgênica Do Milho: Atas, Discussões e Encaminhamentos.

[79] Fernandes G.B., Marinho W. (2017). O Caminho Da Liberalização Dos Transgênicos No Brasil. Agroecología.

[80] CTNBio (2007). Normative Resolution No. 04 of August 16th, 2007. Ministério da Ciência, Tecnologia, Inovações e Comunicação: Brasília, Brazil.

[81] Hofmann F., Otto M., Wosniok W. (2014). Maize pollen deposition in relation to distance from the nearest pollen source under common cultivation-results of 10 years of monitoring (2001 to 2010). Environ. Sci. Eur..

[82] Dyer G.A., Serratos-Hernández J.A., Perales H.R., Gepts P., Piñeyro-Nelson A., Chávez A., Salinas-Arreortua N., Yúnez-Naude A., Taylor J.E., Alvarez-Buylla E.R. (2009). Dispersal of Transgenes through Maize Seed Systems in Mexico. PLoS ONE.

[83] Iversen M., Grønsberg I.M., Berg J.V.D., Fischer K., Aheto D.W., Bøhn T. (2014). Detection of transgenes in local maize varieties of small-scale farmers in Eastern cape, South Africa. PLoS ONE.

[84] Díaz L., Galindo I. (2014). Detección e Identificación de Eventos Asociados a Organismos Vivos Modificados En Semillas de Maíz (*Zea Mays* L.) En Venezuela Empleando Métodos de Inmunoensayo y Análisis Por PCR. Rev. Fac. Agron. UCV.

[85] Kawashima S., Nozaki H., Hamazaki T., Sakata S., Hama T., Matsuo K., Nagasawa A. (2011). Environmental effects on long-range outcrossing rates in maize. Agric. Ecosyst. Environ..

[86] Munarini A., Nerling D., Coelho C.M.M., Nodari R.O. (2021). Maize landraces management to avoid transgenic contamination, decreases yield and seed quality. Bragantia.

[87] CTNBio (2011). Resolução Normativa No 9, de 2 de Dezembro de 2011-REVOGADA PELA RN 24.

[88] CTNBio (2020). Resolução Normativa No 24, de 07 de Janeiro de 2020-REVOGADA PELA RN 32.

[89] CTNBio (2021). Resolução Normativa No 32, de 15 de Junho de 2021.

[90] CTNBio (2015). Resolução Normativa No 15, de 13 de Fevereiro de 2015-REVOGADA PELA RN 24.

[91] Reis J.N.P. (2019). A insustentável distribuição da terra no semiárido brasileiro. Cad. Ciências Sociais Apl..

